# Supplementary material for: Returned Rate and Changed Patterns of Systemic Antibiotic Use in Ambulatory Care in Hungary after the Pandemic—A Longitudinal Ecological Study
Source: Antibiotics (Basel). 2024 Sep 5;13(9):848. doi: 10.3390/antibiotics13090848 (PMC11429041; doi:10.3390/antibiotics13090848)
Supplement: Supplementary file 1 [file antibiotics-13-00848-s001.zip › antibiotics-3144601-supplementary.pdf]

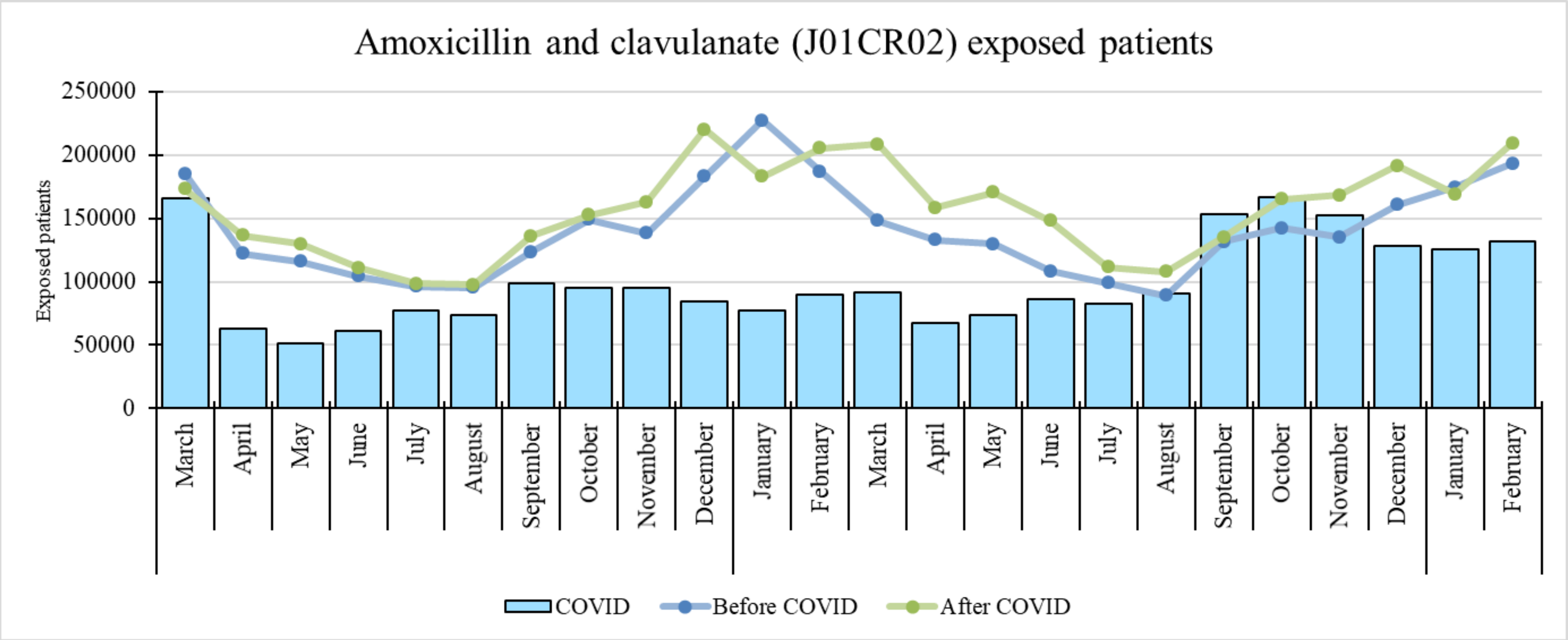

Supplementary Figure S1. Time series of amoxicillin and clavulanate exposed patients

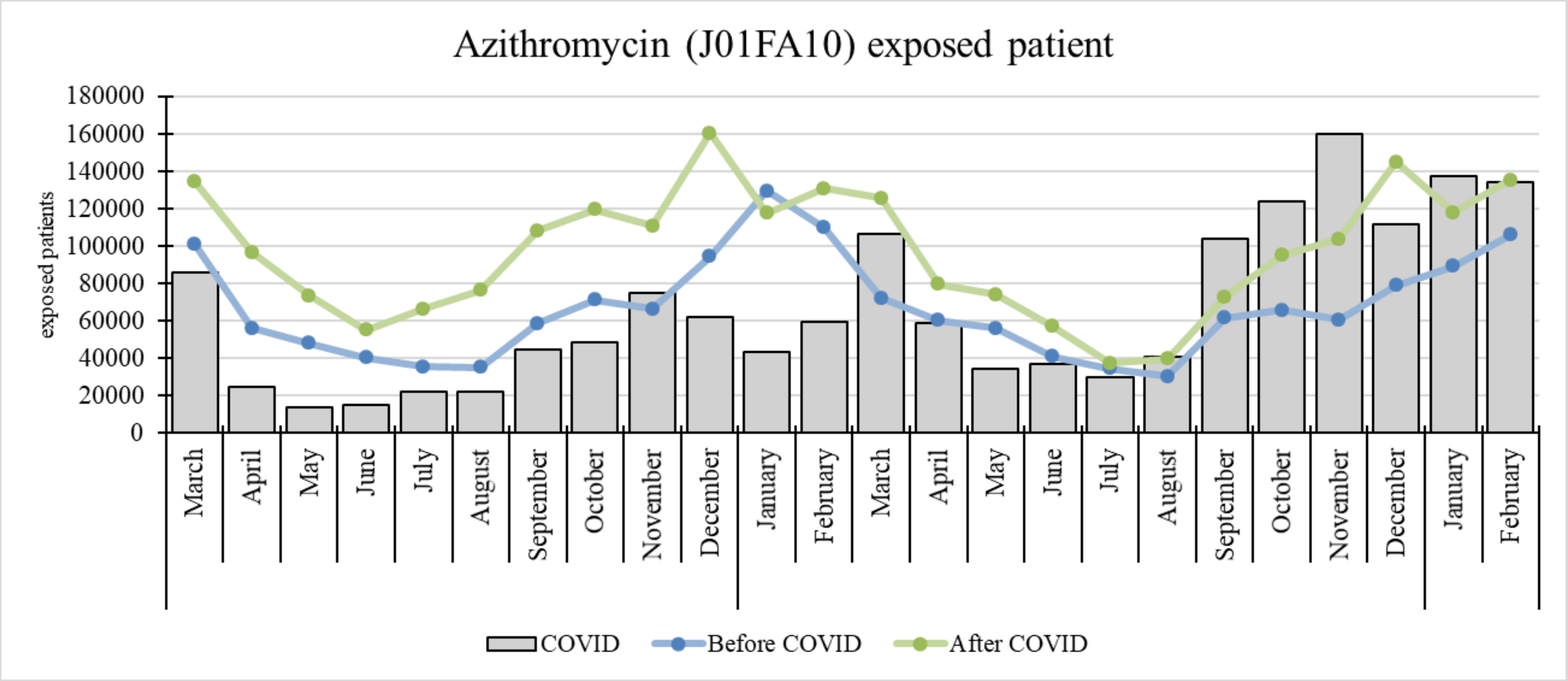

Supplementary Figure S2. Time series of azithromycin exposed patients

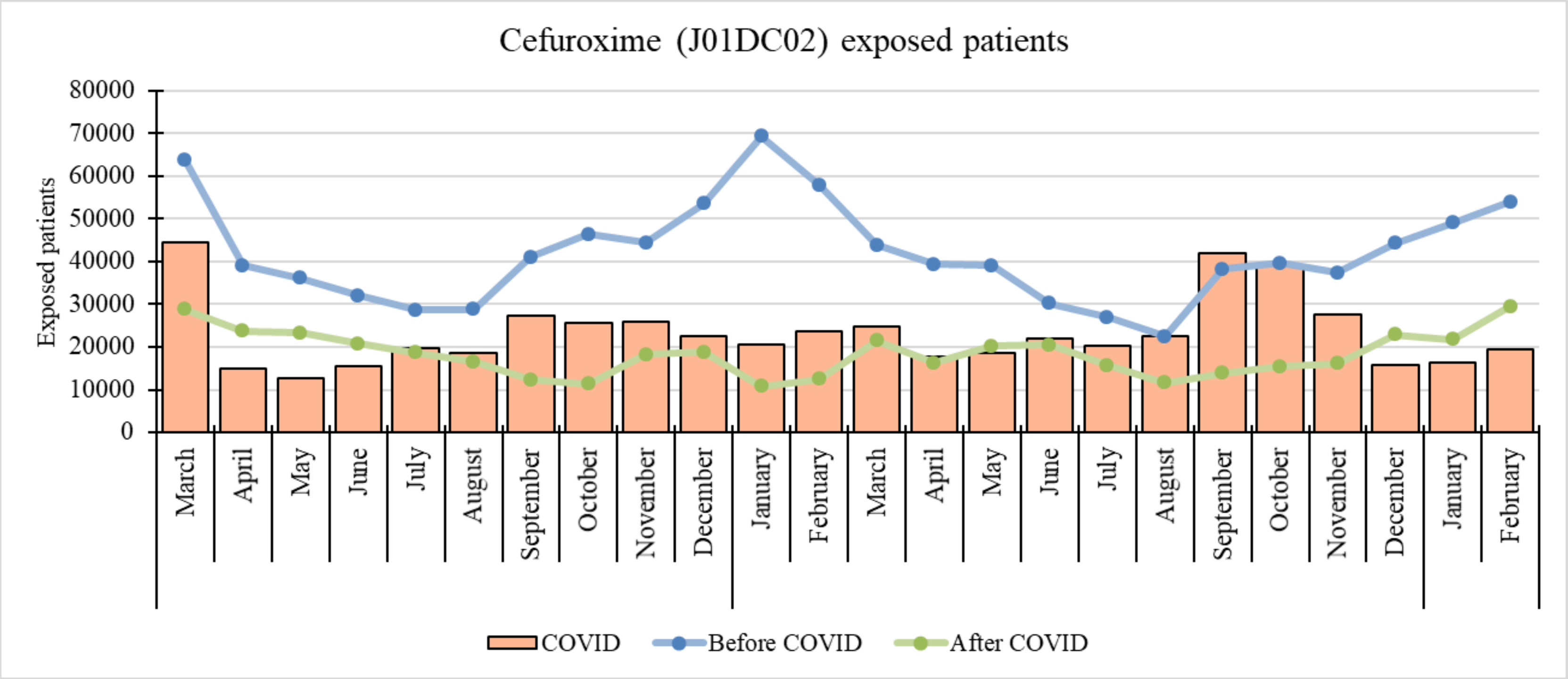

Supplementary Figure S3. Time series of cefuroxime exposed patients

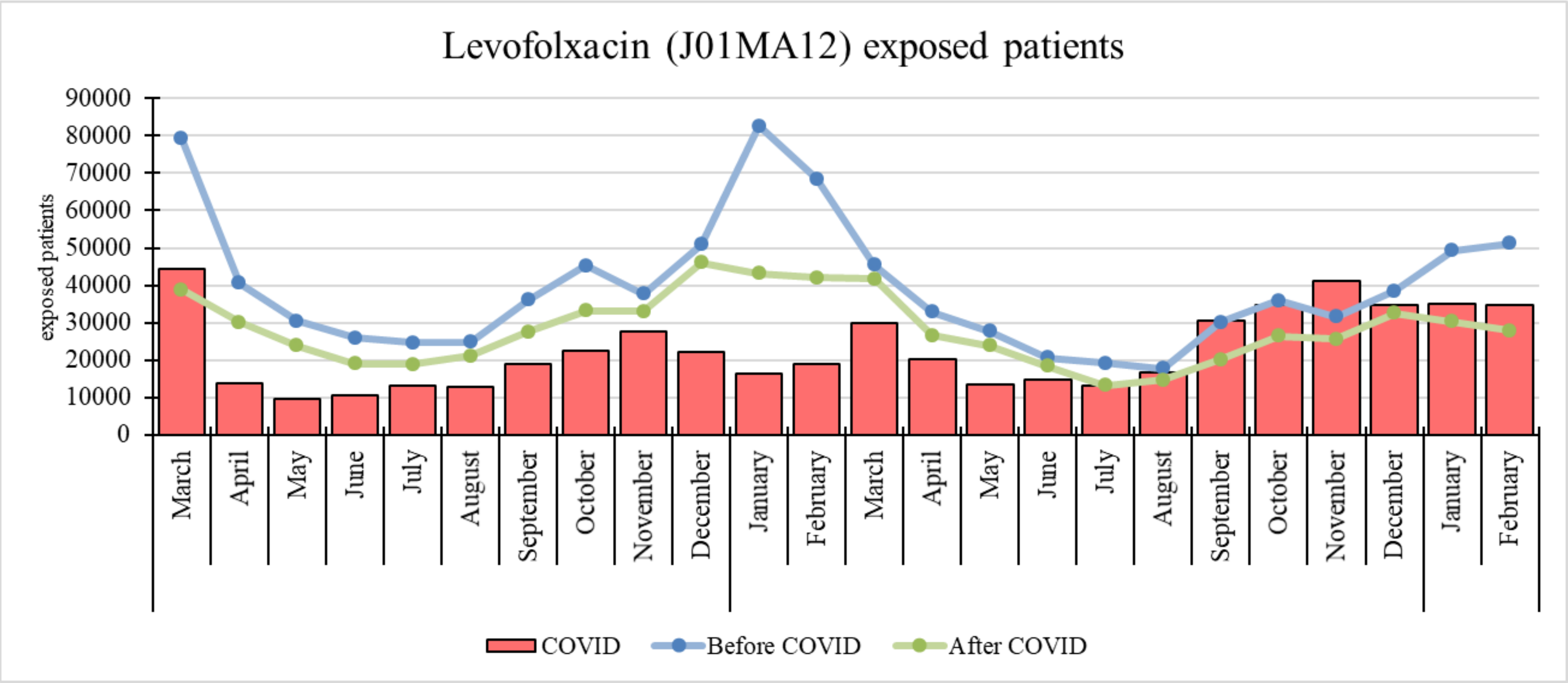

Supplementary Figure S4. Time series of levofloxacin exposed patients

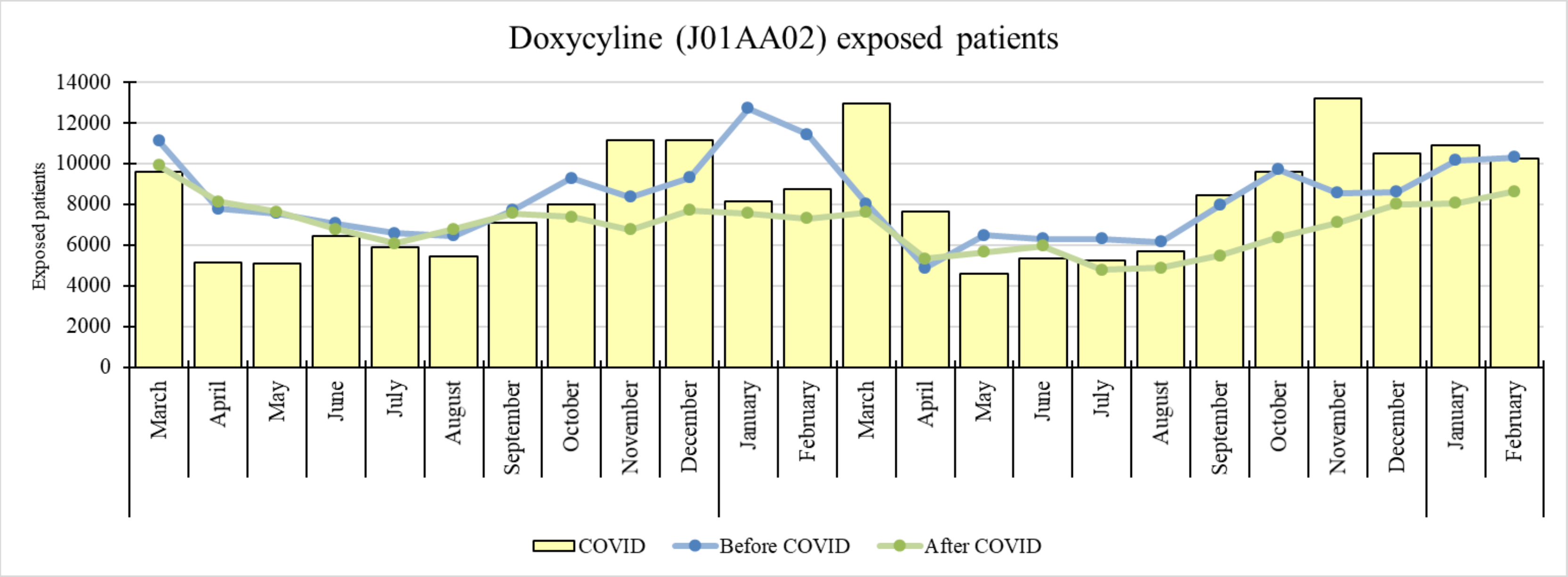

Supplementary Figure S5. Time series of doxycyline exposed patients

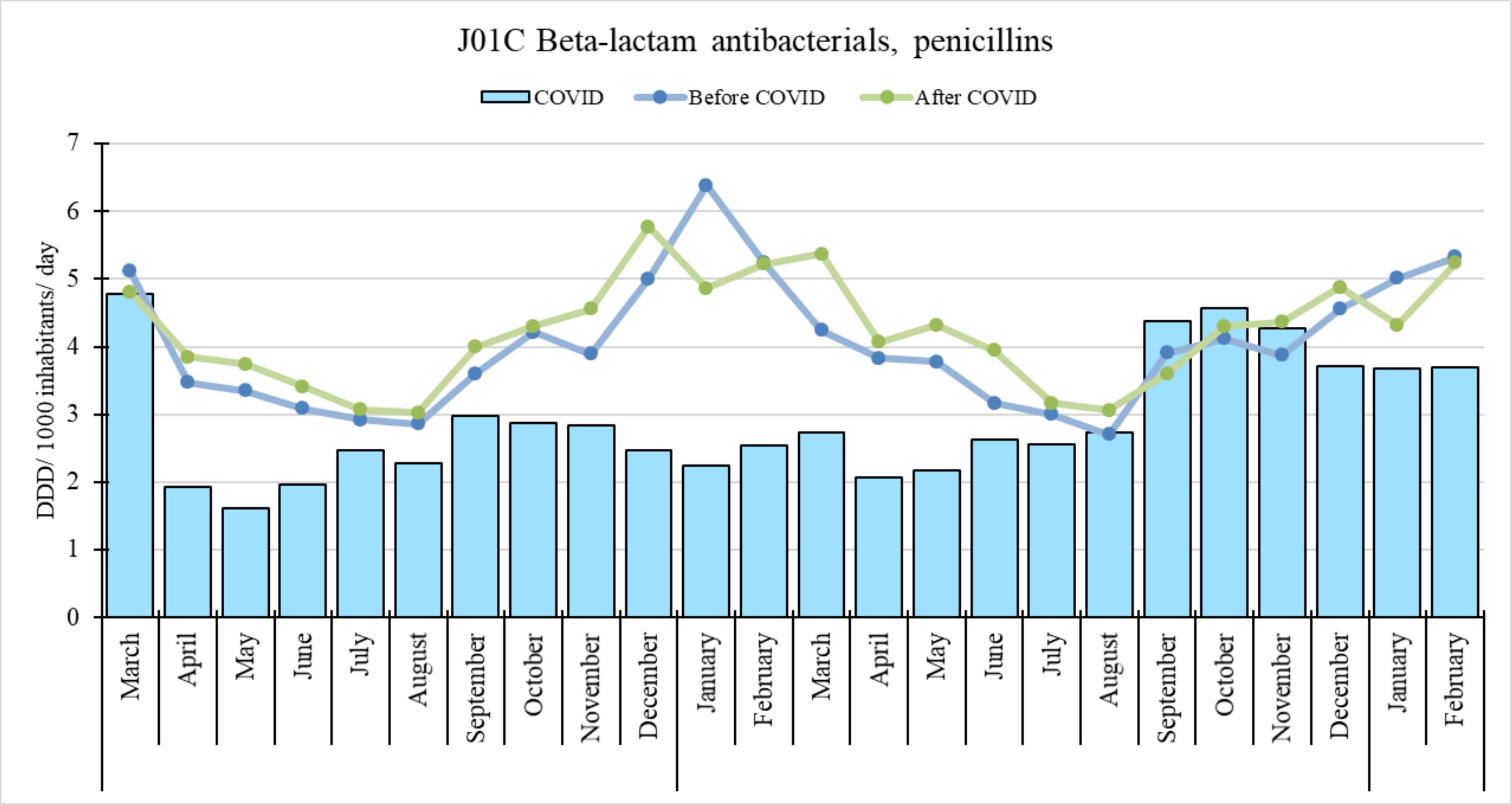

Supplementary Figure S6. Time series of beta-lactam antibacterials, penicillins use expressed as DDD/1000 inhabitants/day

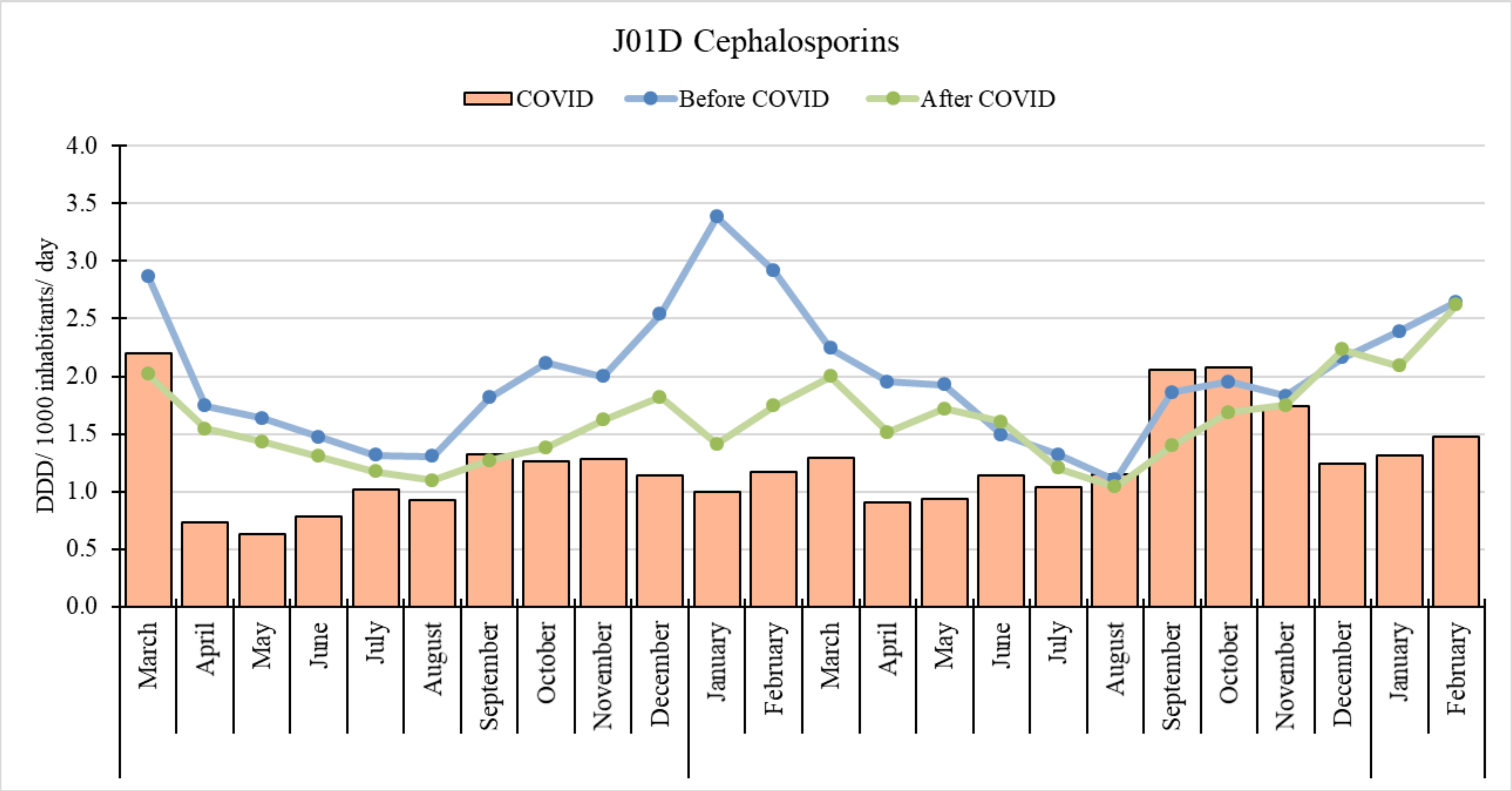

Supplementary Figure S7. Time series of cephalosporins use expressed as DDD/1000 inhabitants/day

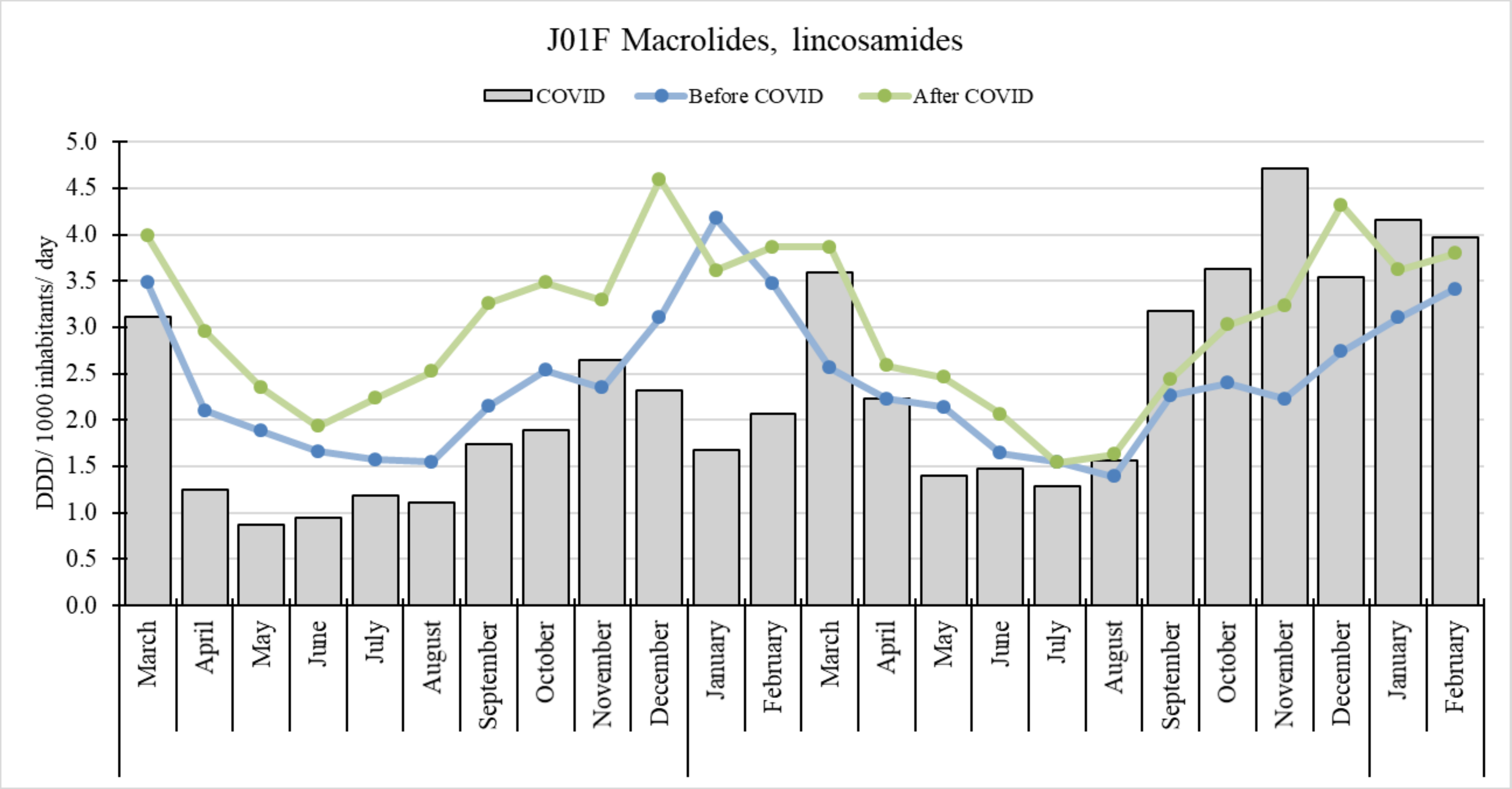

Supplementary Figure S8. Time series of macrolides, lincosamides use expressed as DDD/1000 inhabitants/day

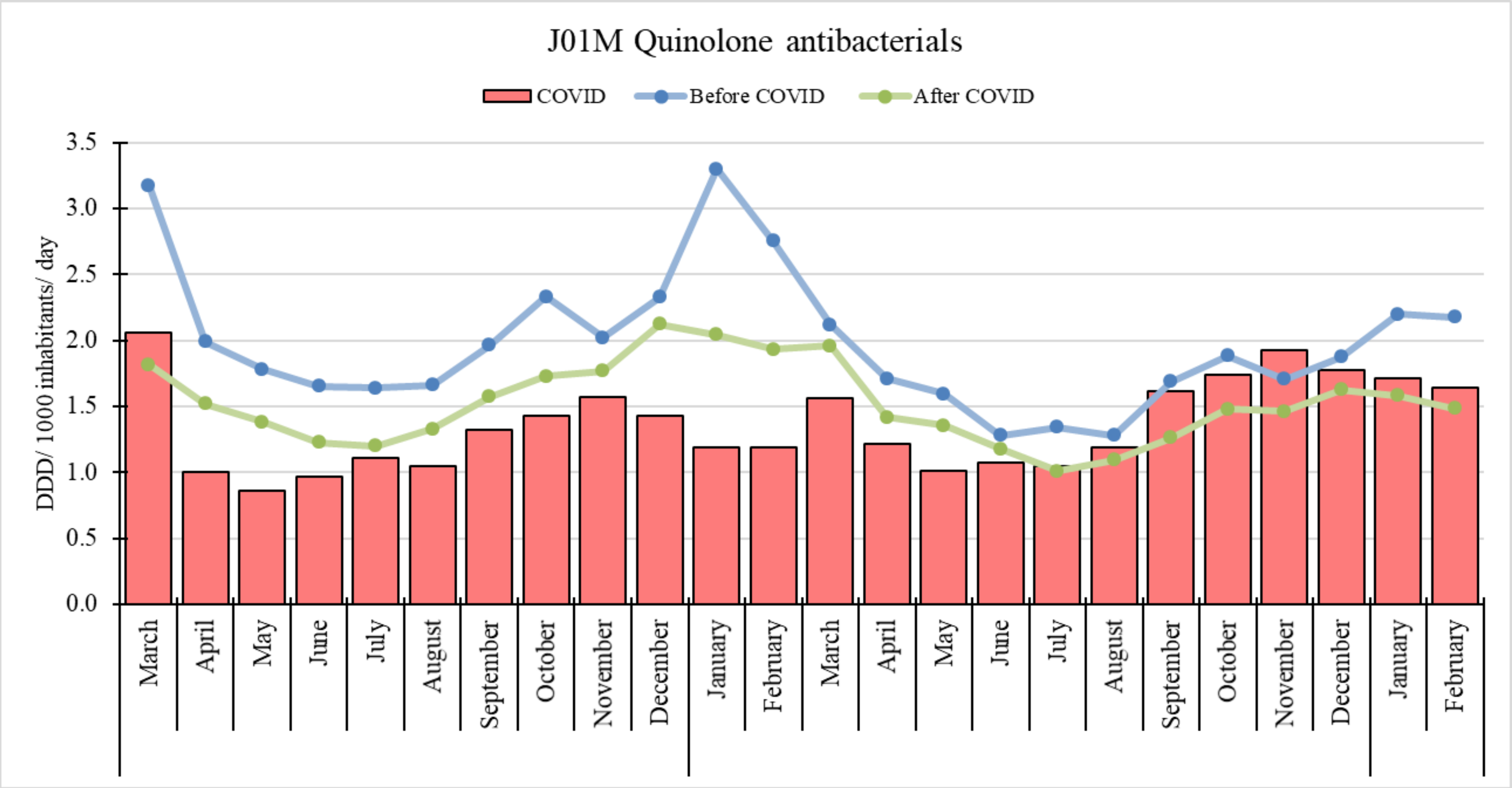

Supplementary Figure S9. Time series of quinolone antibacterials use expressed as DDD/1000 inhabitants/day
